# Identification of a Novel Four-Gene Signature Correlated With the Prognosis of Patients With Hepatocellular Carcinoma: A Comprehensive Analysis

**DOI:** 10.3389/fonc.2021.626654

**Published:** 2021-03-12

**Authors:** Weihua Zhu, Lixin Ru, Zhenchao Ma

**Affiliations:** ^1^ Department of Gastroenterology, Affiliated Huzhou Hospital, Zhejiang University School of Medicine, Huzhou Central Hospital, Affiliated Central Hospital Huzhou University, Huzhou, China; ^2^ Department of Radiation Oncology, Affiliated Huzhou Hospital, Zhejiang University School of Medicine, Huzhou Central Hospital, Affiliated Central Hospital Huzhou University, Huzhou, China; ^3^ Department of Radiation Oncology, The Second Affiliated Hospital of Soochow University, Suzhou, China

**Keywords:** hepatocellular carcinoma, least absolute shrinkage and selection operator Cox regression, weighted gene coexpression network analysis, prognostic signature, single-sample gene set enrichment analysis, immune status

## Abstract

**Purpose:**

Hepatocellular carcinoma (HCC) is a common solid-tumor malignancy with high heterogeneity, and accurate prognostic prediction in HCC remains difficult. This analysis was performed to find a novel prognostic multigene signature.

**Methods:**

The TCGA-LIHC dataset was analyzed for differentially coexpressed genes through weighted gene coexpression network analysis (WGCNA) and differential gene expression analysis. A protein-protein interaction (PPI) network and univariate Cox regression analysis of overall survival (OS) were utilized to identify their prognostic value. Next, we used least absolute shrinkage and selection operator (LASSO) Cox regression to establish a prognostic module. Subsequently, the ICGC-LIRI-JP dataset was applied for further validation. Based on this module, HCC cases were stratified into high-risk and low-risk groups, and differentially expressed genes (DEGs) were identified. Functional enrichment analyses of these DEGs were conducted. Finally, single-sample gene set enrichment analysis (ssGSEA) was performed to explore the correlation between the prognostic signature and immune status.

**Results:**

A total of 393 differentially coexpressed genes were obtained. Forty differentially coexpressed hub genes were identified using the CytoHubba plugin, and 38 of them were closely correlated with OS. Afterward, we established the four-gene prognostic signature with an acceptable accuracy (area under the curve [AUC] of 1-year survival: 0.739). The ICGC-LIRI-JP dataset also supported the acceptable accuracy (AUC of 1-year survival:0.752). Compared with low-risk cohort, HCC cases in the high-risk cohort had shorter OS, higher tumor grades, and higher T stages. The risk scores of this signature still act as independent predictors of OS (P<0.001). Functional enrichment analyses suggest that it was mainly organelle fission and nuclear division that were enriched. Finally, ssGSEA revealed that this signature is strongly associated with the immune status of HCC patients.

**Conclusions:**

The proposed prognostic signature of four differentially coexpressed hub genes has satisfactory prognostic ability, providing important insight into the prediction of HCC prognosis.

## Introduction

It is estimated that nearly 42,810 new cases and 30,160 estimated deaths of hepatocellular carcinoma (HCC) will occur in 2020, leading to enormous socioeconomic pressure for HCC patients and their families (1). HCC accounts for 85%–90% of all primary liver cancer patients, and its occurrence is strongly associated with chronic hepatitis B virus (HBV) or hepatitis C virus (HCV) infection, alcohol consumption, and nonalcoholic steatohepatitis (2). HCC has high interpatient, intertumoral and intratumoral heterogeneity (3). Patients with localized HCC usually have poor survival (with a 5-year overall survival [OS] rate of 30%), and this rate is less than 5% for HCC patients with distant metastasis (4). Currently, due to the complicated etiologic factors and the high heterogeneity of HCC, it remains difficult to accurately predict the prognosis of HCC patients. Although there were some similar studies published previously, they usually required many genes in their gene signatures, which may cause some difficulties in real-world practice (5, 6). Therefore, it is urgent to find the gene signature involved with less genes for the convenience of real-world practice.

With the rapid development of genome technology, bioinformatics analysis has been adopted for microarray datasets to further explore the underlying molecular mechanisms of diseases and detect disease-specific biomarkers (7). Weighted gene coexpression network analysis (WGCNA) is utilized to further understand gene coexpression networks and gene functions (8). WGCNA detects modules of closely correlated genes among samples to relate modules to external traits, providing significant insights into predicting possible functions of coexpressed genes (9). Additionally, differential gene expression analysis is often utilized in transcriptomic datasets to investigate potential biological and molecular mechanisms and quantify differences between the gene expression levels of experimental and control cohorts (10).

To increase the reliability of screening highly related genes, both methods mentioned above were used in our analysis. First, the RNA-Seq dataset and HCC clinical information were downloaded from The Cancer Genome Atlas (TCGA) database. Second, WGCNA and differential gene expression analysis were performed to obtain differentially coexpressed genes. Then, a protein-protein interaction (PPI) network was constructed, and 38 differential coexpression hub genes with prognostic value were detected. Afterward, we built a prognostic four-gene signature and verified it in the International Cancer Genome Consortium (ICGC) database. Ultimately, functional enrichment analysis was conducted to investigate the underlying biological mechanisms.

## Materials and Methods

**Figure 1:**
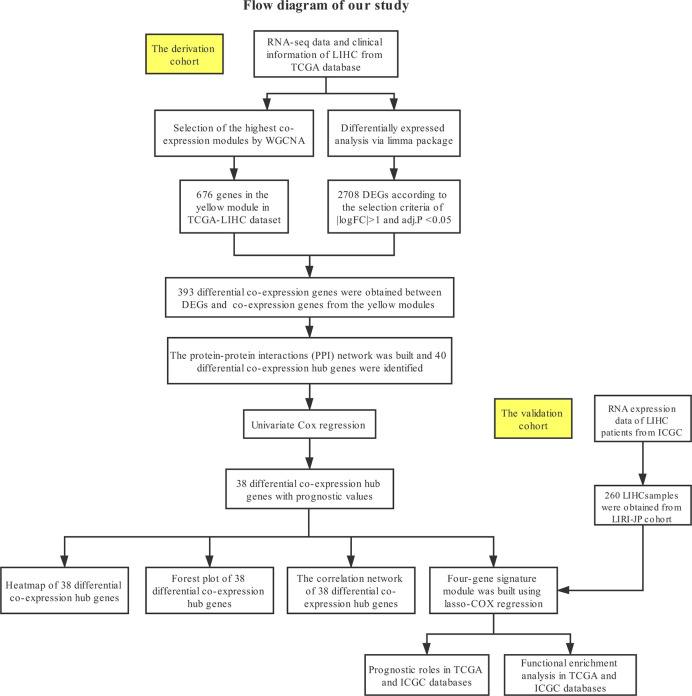
Study design and workflow of this study.

The detailed process of data downloading, prognostic signature construction and external validation is presented in **Figure 1**. The details of each step are illustrated in the following subsections.

### Datasets Downloaded From the TCGA and ICGC Databases

First, RNA-Seq and corresponding clinical data for liver hepatocellular carcinoma (LIHC) were obtained from the TCGA database (https://portal.gdc.cancer.gov/). A list of 424 samples was obtained, including 374 LIHC and 50 normal liver tissues, and RNA-seq count data on 19645 genes were obtained. The Illumina HiSeq 2000 platform was used to generate and annotate all data to a reference transcript set of the human hg38 gene standard track. The edgeR package tutorial suggested that genes with low read counts do not merit further analysis (11). Hence, genes with a count per million (CPM) <1 were omitted from this analysis. Next, the function rpkm in the edgeR package was adapted for further filtering. Consequently, 13,924 genes were acquired for subsequent analysis. Second, the RNA-Seq data and clinical data of HCC patients were acquired from the ICGC database (https://dcc.icgc.org/). A total of 260 HCC samples, which mainly originated from the Japanese population with HBV or HCV infection, were acquired (12). We chose the normalized read count values of the ICGC-LIRI-JP cohort. As a result, 22,913 genes were obtained for the next analysis.

### Identification of Key Coexpression Modules Using WGCNA

**Figure 2:**
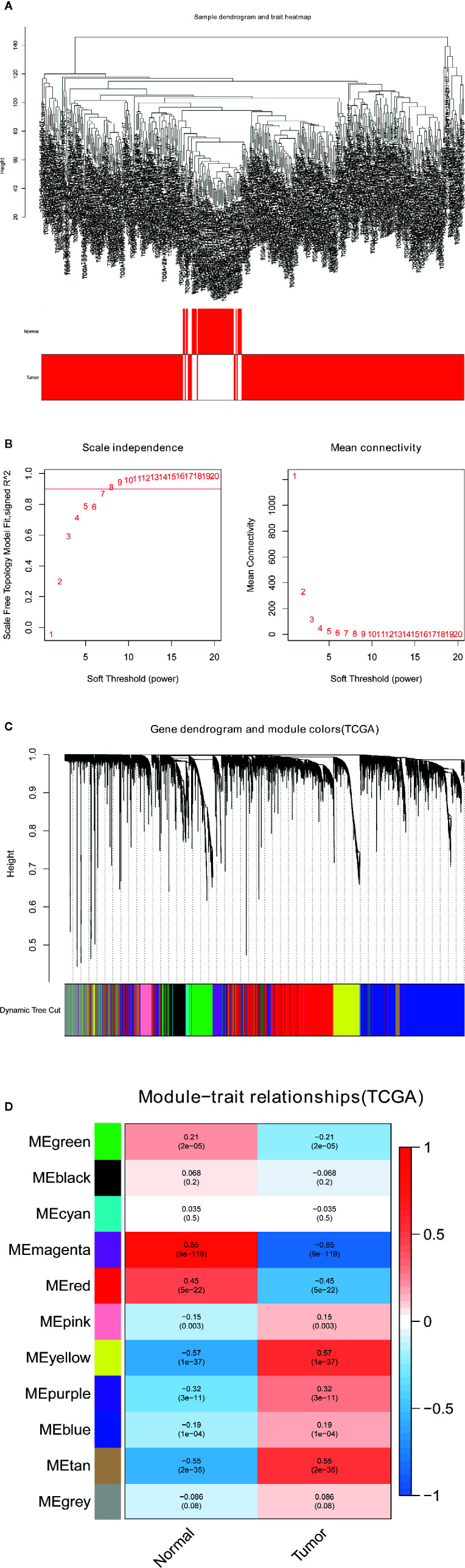
Identification of modules related to the clinical traits in the TCGA-LIHC dataset. **(A)** Sample dendrogram and trait heatmap. **(B)** Scale independence and Mean connectivity. **(C)** The cluster dendrogram of co-expression network modules is ordered by a hierarchical clustering of genes based on the 1-TOM matrix. Different colors represent different modules. **(D)** Module-trait relationships. Each row represents a color module and every column represents a clinical trait (normal and tumor). Each cell contains the corresponding correlation and P-value.

The gene coexpression network of the TCGA-LIHC dataset was built through the WGCNA package (8). To build a scale-free network, a soft-power β = 7 (**Figures 2A, B**) was used in the TCGA-LIHC dataset. Next, the adjacency matrix was created according to the formula aij = |Sij|^β^ (aij: adjacency matrix between gene i and gene j, Sij: similarity matrix made by Pearson’s correlation coefficient of all gene pairs, as well as β: soft-power value). Subsequently, we converted this matrix into a topological overlap matrix (TOM) and the corresponding dissimilarity (1-TOM). The hierarchical clustering dendrogram of the 1-TOM matrix was established to aggregate the genes with similar expression patterns into the same coexpression module. Afterward, the module-trait relations between modules and external traits were analyzed to identify functional modules from the coexpression network. Hence, the modules with the largest correlation coefficients were regarded as modules that highly correlated with clinical traits. We chose the module that was positively associated with LIHC for our subsequent analysis.

### Identification of Differentially Coexpressed Genes

The limma package is often used to perform differential gene expression analysis of gene expression profiles and RNA-Seq datasets (13). Here, we applied the limma package in the differential expression analysis of the TCGA-LIHC dataset to identify differentially expressed genes (DEGs) between LIHC and nontumorous tissues. To minimize the false discovery rate (FDR) to the greatest extent possible, we adjusted the P-value with the Benjamini–Hochberg (BH) method. The filtering criteria for DEGs were |logFC|>1 and adj. P <0.05. Afterward, we took the intersection of genes between DEGs and coexpressed genes to improve the reliability of screening closely related genes, and these differentially coexpressed genes were used for the next analysis.

### PPI Network Construction and Hub Gene Identification

The PPI network of differentially coexpressed genes was built through the Search Tool for the Retrieval of Interacting Genes (STRING) database (14). Then, we established a visual network of molecular interactions with combined scores ≥0.7 using Cytoscape (15). In addition, the degree values of all nodes in the PPI network were calculated using the CytoHubba plugin (16). The top 40 nodes with the highest degree scores were selected and regarded as hub genes associated with LIHC. The forty hub genes related to LIHC were displayed using the CytoHubba plug-in. In addition, we conducted gene ontology (GO) and Kyoto Encyclopedia of Genes and Genomes (KEGG) pathway analyses of the 40 hub genes to explore their biological functions. Adj. P values <0.05 were considered significant.

### Survival Analysis of Hub Genes and the Correlation Network

To analyze the prognostic roles of the differentially coexpressed hub genes in LIHC, we performed univariate Cox regression analysis of OS using the survival package based on the TCGA-LIHC dataset. LIHC patients without follow-up information or a survival time=0 days were excluded from our analysis, and the other patients in the TCGA-LIHC dataset were classified into two groups considering the median expression levels of the differentially coexpressed hub genes. Log-rank P<0.01 was considered significant. Additionally, the correlation network of these differentially coexpressed hub genes was established through the igraph package. The filtering criterion was a cutoff >0.75.

### Construction of the Gene Signature in the TCGA Database

To decrease the risk of overfitting to the greatest extent possible, we used least absolute shrinkage and selection operator (LASSO) Cox regression analysis to build the prognostic module of LIHC (17, 18). The LASSO algorithm is widely utilized to select and shrink variables using the glmnet package. We used the expression matrix of the differentially coexpressed hub genes with prognostic value as the independent variable, while the OS and status of patients in the TCGA-LIHC dataset were used as the response variables. Then, we determined the penalty parameter (λ) of this module using tenfold cross-validation following the minimum criteria, namely, the λ value corresponding to the minimum partial likelihood deviance.

### Nomogram and Validation of the Expression Patterns of the Gene Signature

We calculated the risk scores of all LIHC patients using the expression level of every gene and the corresponding regression coefficient. The following formula was used: score= e^sum (every gene’s expression × corresponding coefficient)^. Then, LIHC patients were divided into high- and low-risk cohorts based on the median value of the risk score. Subsequently, we constructed a nomogram of the prognostic signature to predict the survival of LIHC patients. Furthermore, we built calibration curves and time-dependent receiver operating characteristic (ROC) curves to evaluate the discrimination and accuracy of the prognostic multigene signature. The GSE112790 dataset was used to validate the expression patterns of the genes in the signature between LIHC and nontumorous tissues.

### Distribution and Prognostic Value of the Gene Signature

To analyze the prognostic value of the gene signature, we performed Kaplan-Meier survival analysis between the low- and high-risk groups using the survminer package based on the TCGA-LIHC and ICGC-LIRI-JP datasets. Additionally, to explore distribution in the low- and high-risk cohorts, we performed principal component analysis (PCA) and t-distributed stochastic neighbor embedding (t-SNE) on the TCGA-LIHC and ICGC-LIRI-JP datasets using the stats and Rtsne packages, respectively. To determine whether the risk score acts as an independent indicator of the prognosis of LIHC patients, we performed univariate and multivariate Cox regression analyses among all available variables using the TCGA-LIHC and ICGC-LIRI-JP datasets.

### Differential Gene Expression Analysis and Functional Enrichment Analysis

To acquire the DEGs between the low- and high-risk groups, we performed differential gene expression analysis using the limma package in the TCGA-LIHC and ICGC-LIRI-JP datasets. The P-value was adjusted using the BH method. The filtering criteria for DEGs were |logFC|>2 and adj. P <0.05. Afterward, we conducted GO and KEGG pathway analyses of the DEGs between the low- and high-risk groups in the TCGA-LIHC and ICGC-LIRI-JP datasets. To further analyze the relationship between the risk score and immune status, we calculated the infiltrating scores of 16 immune cells and 13 immune-related functions or pathways using single-sample gene set enrichment analysis (ssGSEA) (19).

## Results

### Identification of Key Coexpression Modules Using WGCNA

To find the pivotal module in LIHC, the gene coexpression network was established in the TCGA-LIHC dataset. A list of 11 modules was generated (**Figure 2C**). Next, the heatmap revealed the correlations between the modules and clinical traits (normal and LIHC) in the TCGA-LIHC dataset (**Figure 2D**). Furthermore, the yellow module of the TCGA-LIHC dataset positively correlated with LIHC tissues (r=0.57, P=1e-37) and was used for our next analysis.

### Selection of Differentially Coexpressed Genes

**Figure 3:**
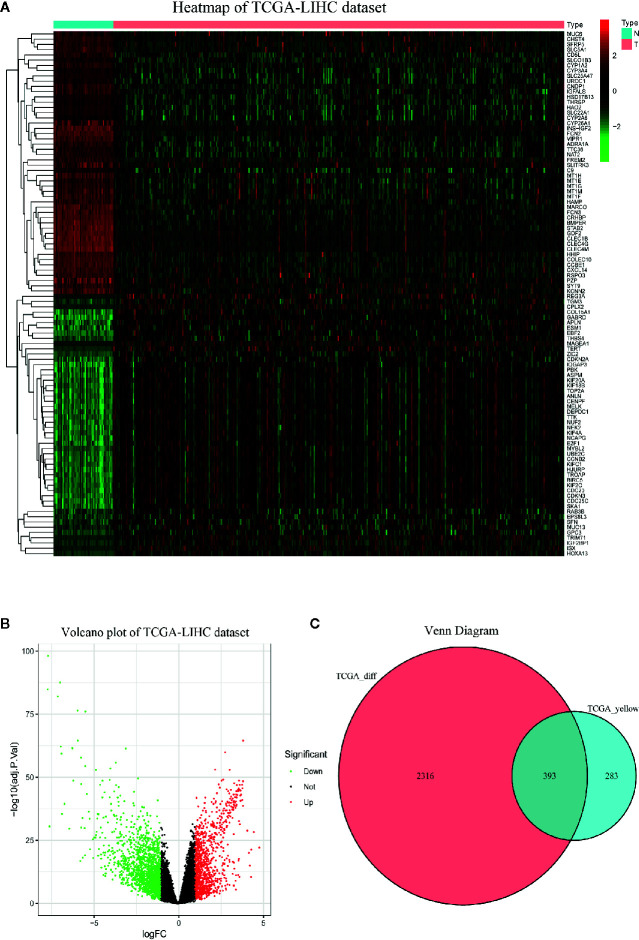
Identification of differentially expressed genes (DEGs) in TCGA-LIHC dataset with the cut-off criteria of |logFC|>1 and adj.P <0.05. **(A)** Heatmap of top 50 upregulated and 50 downregulated DEGs of TCGA-LIHC dataset. **(B)** Volcano plot of DEGs in the TCGA-LIHC dataset. **(C)** The Venn diagram of genes between DEGs and co-expression genes. A total of 393 overlapping differential co-expression genes are detected.

The heatmap displayed the expression patterns of fifty upregulated and fifty downregulated genes in the TCGA-LIHC dataset (**Figure 3A**). The volcano plot indicated that 2708 DEGs had a conspicuous dysregulation between LIHC and nontumorous tissues in the TCGA-LIHC dataset (**Figure 3B**). The Venn diagram showed the intersection of coexpressed genes (**Table S1**) and DEGs (**Table S2**); namely, 393 differentially coexpressed genes were identified (**Figure 3C**).

### PPI Network Construction and Hub Gene Analysis

**Figure 4:**
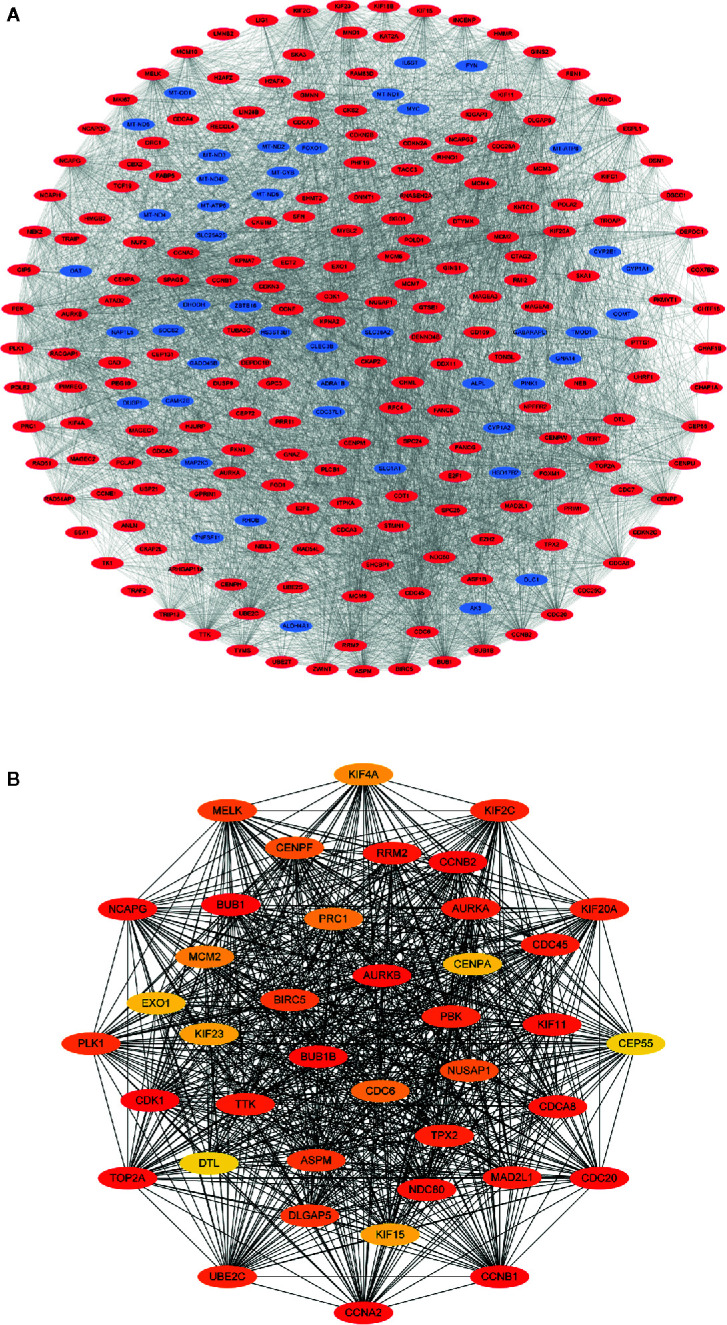
Visualization of the protein-protein interaction (PPI) network and hub genes. **(A)** PPI network of differential co-expression genes. **(B)** The identification of 40 differential co-expressed hub genes using the degree algorithm.

**Figure 4A** displays the PPI network of the differentially coexpressed genes with 241 nodes and 4792 edges. Subsequently, we quantified the degree scores of all nodes in this PPI network through the CytoHubba plugin (**Table S3**) and chose the top 40 nodes as hub genes that are closely correlated with LIHC (**Figure 4B**). In addition, GO analysis showed significant enrichment in the mitotic nuclear division, organelle fission and spindles terms (**Figure S1A**). KEGG pathway analysis showed enrichment in the cell cycle and oocyte meiosis pathways (**Figure S1B**).

### Survival Analysis and Correlation Network of the Differentially Coexpressed Hub Genes

**Figure 5:**
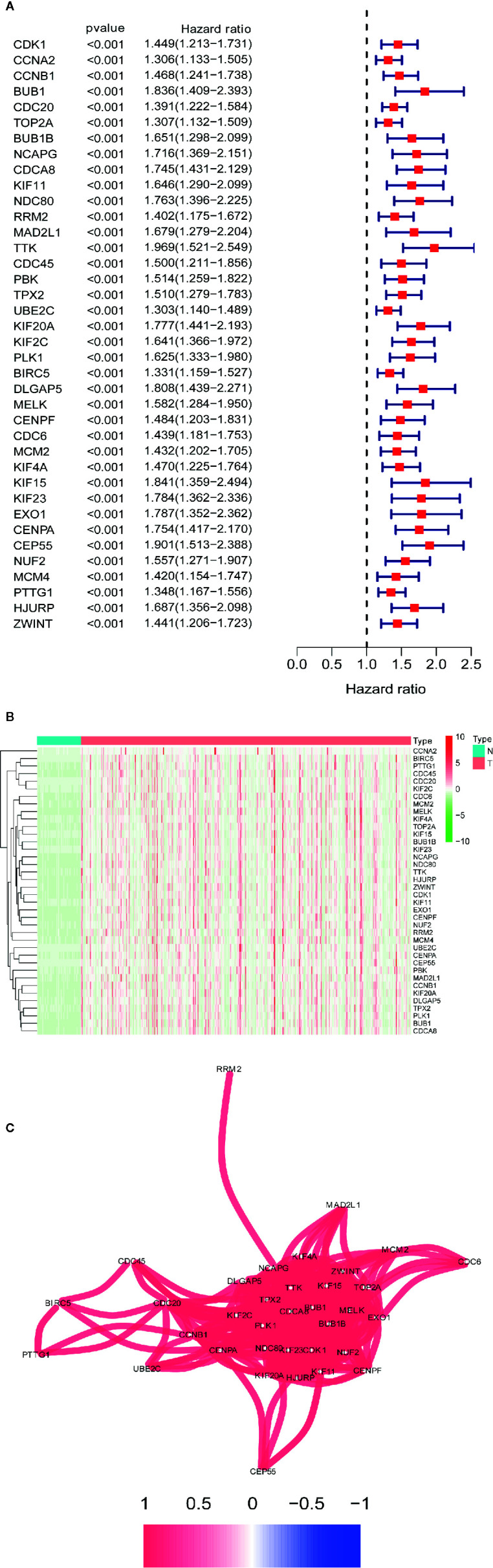
Identification differential co-expressed hub genes with prognostic values. **(A)** Univariate Cox analysis for overall survival (OS) of 38 differential co-expressed hub genes with prognostic values. **(B)** 38 differential co-expressed hub genes with prognostic values are significantly upregulated in HCC tissues. **(C)** The correlation network of candidate genes. The correlation coefficients are represented by different colors.

Univariate Cox regression analysis of the differentially coexpressed hub genes demonstrated that 38 hub genes were closely associated with the survival of LIHC patients (**Figure 5A**). The heatmap revealed that the 38 hub genes with prognostic value were significantly overexpressed in LIHC tissues (**Figure 5B**). Additionally, the correlation network suggested that the differentially coexpressed hub genes closely interact with each other (**Figure 5C**).

### Construction of the Gene Signature and Nomogram in the TCGA Database

**Figure 6:**
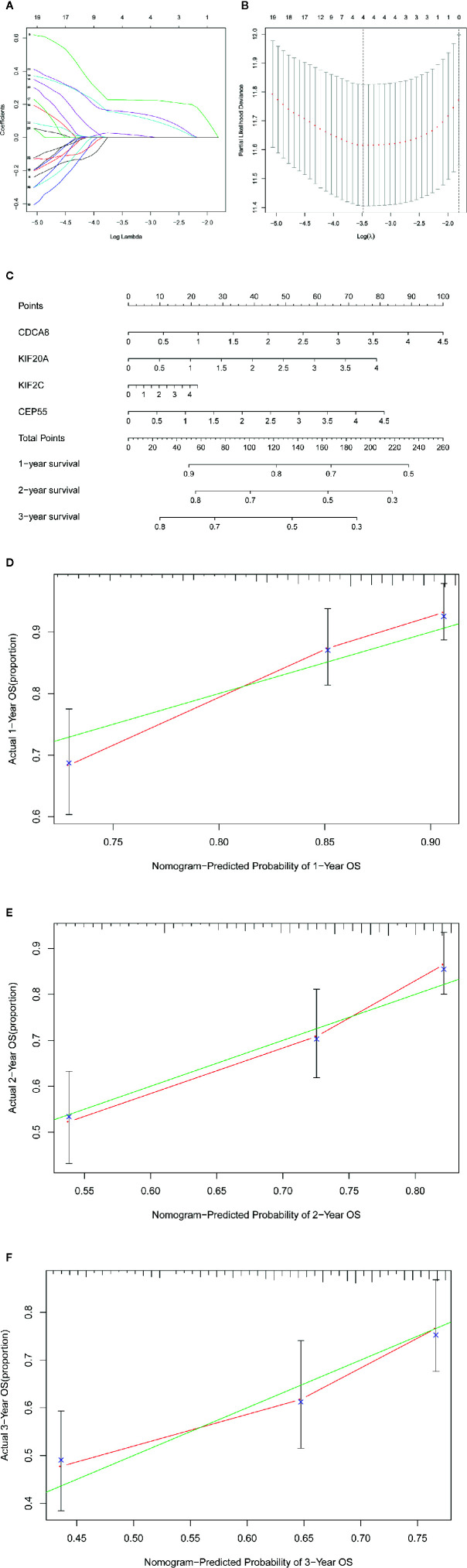
|Construction of the gene signature and nomogram in TCGA-LIHC dataset. **(A, B)** The construction of the four-gene signature module. **(C)** The construction of the nomogram of this module. **(D–F)** The calibration curves of 1-, 2-, and 3-year overall survival probability.

**Figure 7:**
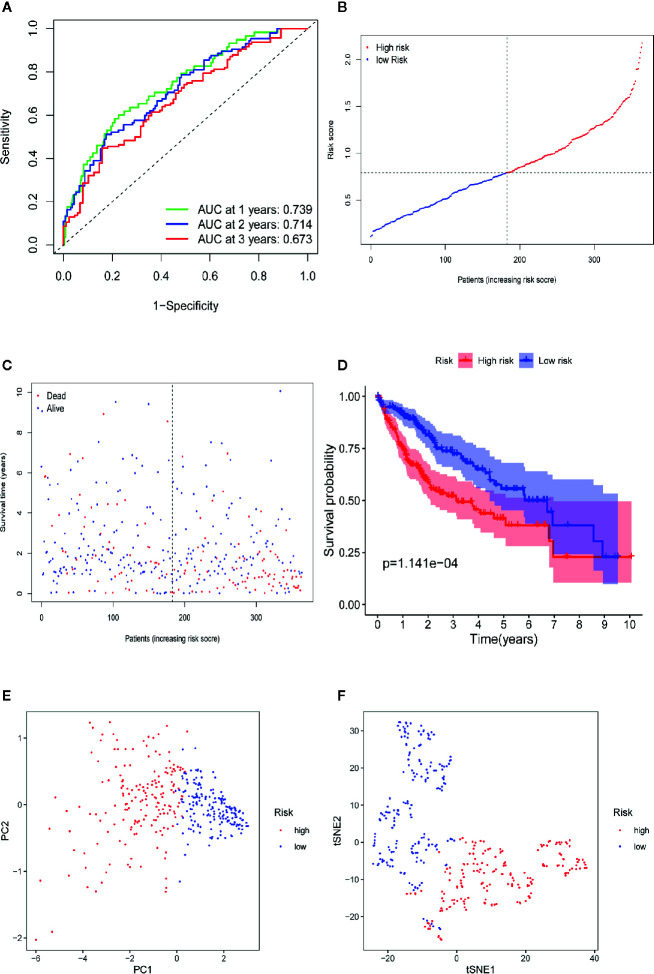
Prognostic analysis of the four-gene signature model in TCGA-LIHC dataset. **(A)** AUC of time-dependent ROC curves verifies the prognostic performance of the risk score in TCGA-LIHC dataset. **(B)** The distribution and the median value of the risk scores in TCGA-LIHC dataset. **(C)** The distributions of OS status, OS and risk score in the TCGA-LIHC dataset. **(D)** Kaplan-Meier curves for the OS of patients in the high-risk group and low-risk group in TCGA-LIHC dataset. **(E)** PCA plot of TCGA-LIHC dataset. **(F)** t-SNE analysis of TCGA-LIHC dataset.

We used the LASSO Cox regression module to build a prognostic signature based on the expression matrix of the 38 differentially coexpressed hub genes. Consequently, we identified a four-gene signature module according to the optimal λ value (**Figures 6A, B**). In addition, we calculated the risk scores of LIHC patients using the following formula: score= e ^(0.225*expression value of CDCA8+0.124*expression value of KIF20A+0.012*expression value of KIF2C+0.144*expression value of CEP55)^. Then, we established a nomogram to predict the 1-, 2-, and 3-year OS probability of LIHC patients (**Figure 6C**). The calibration curves of 1-, 2-, and 3-year OS probability showed satisfactory calibration of this nomogram (**Figures 6D–F**). Moreover, based on GSE112790, we confirmed that CDCA8, KIF20A, KIF2C and CEP55 were significantly overexpressed in LIHC tissues compared with nontumorous tissues (**Figure S2**). Furthermore, the ROC curves suggested acceptable accuracy of this nomogram (area under the curve [AUC] of 1-year survival: 0.739; AUC of 2-year survival: 0.714; and AUC of 3-year survival: 0.673) (**Figure 7A**). Afterward, all LIHC patients were divided into a low-risk cohort (n=183) and a high-risk cohort (n=182) based on the median risk score (**Figure 7B**). The high-risk cohort in the TCGA-LIHC dataset had more deaths (**Figure 7C**), a poorer tumor grade, a higher clinical stage and a higher T stage (**Table 1**). Consistently, Kaplan-Meier survival analysis showed that LIHC patients in the high-risk cohort experienced shorter survival than those in the low-risk cohort (**Figure 7D**, P=1.14e-4). In the PCA of the TCGA-LIHC dataset, the first principal component (PC1) could explain 88.6% of total variance, and the PC1 scores were negatively correlated with the risk scores of patients (**Figure 7E**), while the second principal component (PC2) could explain 5.4% total variance (**Figure S3A**). Moreover, PCA and t-SNE analysis revealed that most LIHC patients in the high- and low-risk cohorts were distributed in two different directions (**Figure 7F**).

**Table 1:** Baseline characteristics of LIHC patients in high-risk and low-risk cohorts.

Baseline characteristics		TCGA-LIHC dataset			ICGC-LIRP-JI dataset		
		High-risk	Low-risk	P-value	High-risk	Low-risk	P-value
Age (%)	≤60 year	97 (53.3)	76 (41.5)	**0.024**	35 (21.5)	15 (21.7)	0.964
	>60 year	85 (46.7)	105 (58.5)		128 (78.5)	54 (78.3)	
Gender (%)	Female	66 (36.3)	53 (29.0)	0.137	44 (27.2)	17 (24.6)	0.691
	Male	116 (63.7)	130 (71.0)		118 (72.8)	52 (75.4)	
Tumor grade (%)	G1 + G2	94 (51.6)	136 (74.3)	**<0.001**	–	–	–
	G3 + G4	85 (46.7)	45 (24.6)		–	–	
	unknown	3 (1.6)	2 (1.1)		–	–	
Clinical stage (%)	I + II	116 (63.7)	138 (75.4)	**0.008**	–	–	–
	III + IV	54 (29.7)	33 (18.0)		–	–	
	unknown	12 (6.6)	12 (6.6)		–	–	
T stage (%)	T1 + T2	124 (68.1)	147 (80.3)	**0.003**	96 (59.3)	45 (65.2)	0.395
	T3 + T4	58 (31.9)	33 (18.0)		66 (40.7)	24 (34.8)	
	unknown	0 (0.0)	3 (1.7)		0 (0.0)	0 (0.0)	
N stage (%)	N0	127 (69.8)	121 (66.1)	0.962	–	–	–
	N1 + N2 + N3	2 (1.1)	3 (1.6)		–	–	
	unknown	53 (29.1)	53 (29.0)		–	–	
M stage (%)	M0	136 (74.7)	127 (69.4)	0.230	–	–	–
	M1	0 (0.0)	3 (1.6)		–	–	
	unknown	46 (25.3)	53 (29.0)		–	–	

LIHC, liver hepatocellular carcinoma; TCGA, The Cancer Genome Atlas; ICGC, International Cancer Genome Consortium. The bold P values means P < 0.05.

### Verification of the Four-Gene Signature Module in the ICGC Database

**Figure 8:**
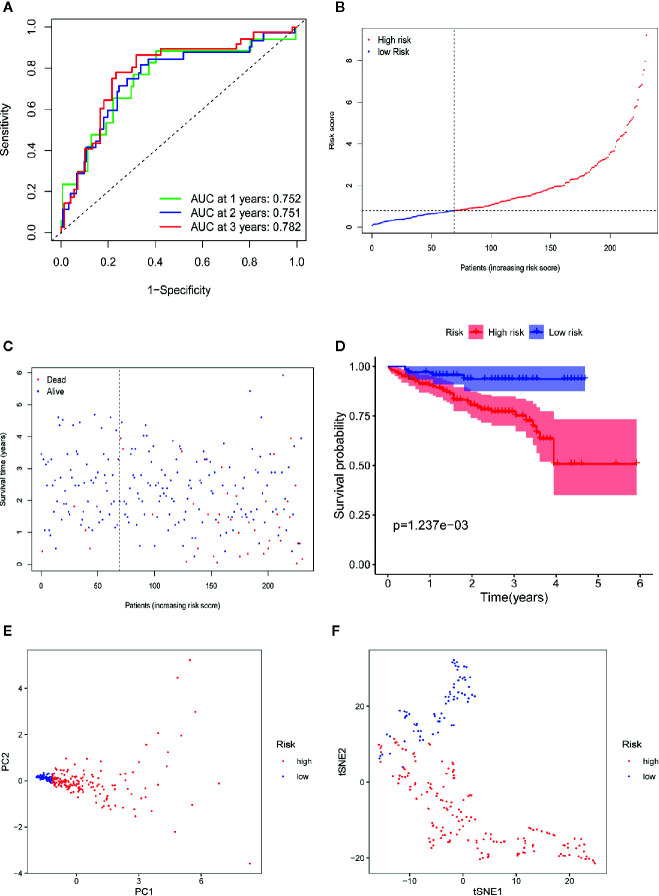
Validation of the 10-gene signature in ICGC-LIRI-JP dataset. **(A)** AUC of time-dependent ROC curves verifies the prognostic performance of the risk score in ICGC-LIRI-JP dataset. **(B)** The distribution and the median value of the risk scores in ICGC-LIRI-JP dataset. **(C)** The distributions of OS status, OS and risk scores in ICGC-LIRI-JP dataset. **(D)** Kaplan-Meier curves for the OS of patients in the high-risk group and low-risk group in ICGC-LIRI-JP dataset. **(E)** PCA plot of ICGC-LIRI-JP dataset. **(F)** t-SNE analysis of ICGC-LIRI-JP dataset.

To validate the robustness of the four-gene signature module from the TCGA-LIHC dataset, we chose the ICGC-LIRI-JP dataset for further verification. First, we stratified LIHC patients from the ICGC-LIRI-JP dataset into high-risk and low-risk cohorts according to the median value of the risk score, which was calculated using the formula mentioned above. Consistent with the outcomes from the TCGA-LIHC dataset, the four-gene signature had an excellent AUC (**Figure 8A**, 1-year survival: 0.752; 2-year survival: 0.751; and 3-year survival: 0.782). Moreover, the high-risk group correlated with a higher rate of mortality (**Figures 8B, C**). Additionally, patients from the high-risk cohort experienced significantly shorter survival than those in the low-risk cohort (**Figure 8D**, P=1.24e-3). In the PCA of the ICGC-LIRI-JP dataset, the PC1 could explain 79% of total variance, and the PC1 scores were positively correlated with the risk scores of patients (**Figure 8E**), whereas the PC2 could explain 13% total variance (**Figure S3B**). In addition, t-SNE analysis validated that most patients in the high- and low-risk cohorts were distributed in two different directions (**Figure 8F**). In general, these outcomes in the ICGC-LIRI-JP dataset were similar to those in the TCGA-LIHC dataset.

### Independent Prognostic Role of the Four-Gene Signature

**Figure 9:**
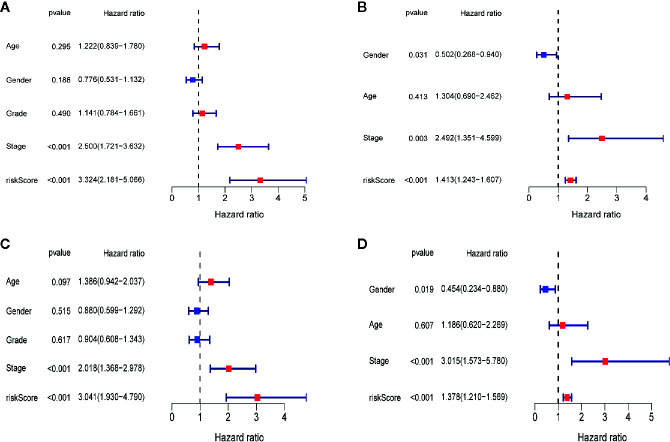
Independent prognostic role of the four-gene signature. **(A)**The univariate Cox regression analysis in TCGA-LIHC dataset. **(B)**The univariate Cox regression analysis in ICGC-RI-JP dataset. **(C)** The multivariate Cox regression analysis in TCGA-LIHC dataset. **(D)** The multivariate Cox regression analysis in ICGC-LIRI-JP dataset.

To determine whether the risk score plays an independent prognostic role, we performed univariate and multivariate Cox regression analyses of the survival of LIHC patients. The univariate Cox regression analysis indicated that a higher risk score was closely correlated with worse survival in LIHC patients using the TCGA-LIHC (**Figure 9A**, hazard ratio [HR]=3.324, 95% confidence interval [CI]: 2.181–5.066, P<0.001) and ICGC-LIRI-JP (**Figure 9B**, HR=1.413, 95% CI: 1.243–1.607, P<0.001) datasets. Similar to the results of the univariate Cox regression analysis, the multivariate Cox regression analysis still suggested the risk score as an independent indicator for the survival of LIHC patients using the TCGA-LIHC (**Figure 9C**, HR=3.041, 95% CI: 1.930–4.790, P<0.001) and ICGC-LIRI-JP (**Figure 9D**, HR=1.378, 95% CI: 1.210–1.569, P<0.001) datasets.

### Differential Gene Expression Analysis and Functional Enrichment Analysis

**Figure 10:**
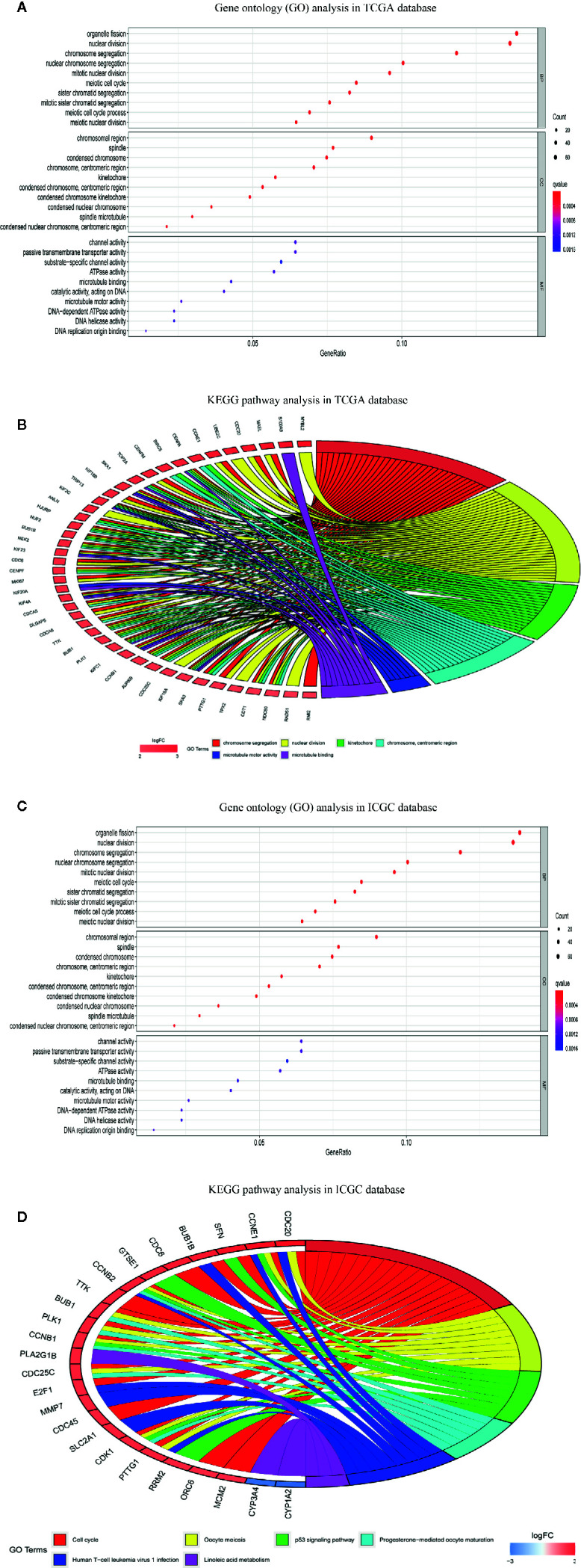
Functional enrichment analysis of differentially expressed genes (DEGs) between high-risk and low-risk groups. **(A)** Gene ontology (GO) enrichment analysis of DEGs of TCGA-LIHC dataset. **(B)** Gene ontology (GO) enrichment analysis of DEGs of ICGC-RI-JP dataset. **(C)** Kyoto encyclopedia of genes and genomes pathway analysis of DEGs of TCGA-LIHC dataset. **(D)** KEGG pathway analysis of DEGs of ICGC-RI-JP dataset.

Differential gene expression analyses were conducted in the TCGA-LIHC and ICGC-LIRI-JP datasets, and 499 and 185 DEGs between the high- and low-risk groups were obtained (**Tables S4**, **S5**), respectively. To explore the biological functions of the DEGs in the high- and low-risk groups, we again performed GO enrichment and KEGG pathway analyses. In the TCGA-LIHC dataset, GO enrichment analysis indicated significant enrichment in the organelle fission, nuclear division, chromosomal region and ATPase activity terms (**Figure 10A**). GO enrichment analysis of the ICGC-LIRI-JP dataset showed similar outcomes to the TCGA-LIHC dataset (**Figure 10B**). Additionally, KEGG pathway analysis of the TCGA-LIHC dataset showed significant enrichment in the cell cycle, oocyte meiosis and progesterone-medicated oocyte maturation pathways (**Figure 10C**). In the ICGC-LIRI-JP dataset, KEGG pathway analysis also demonstrated the analogical outcomes of the TCGA-LIHC dataset (**Figure 10D**).

**Figure 11:**
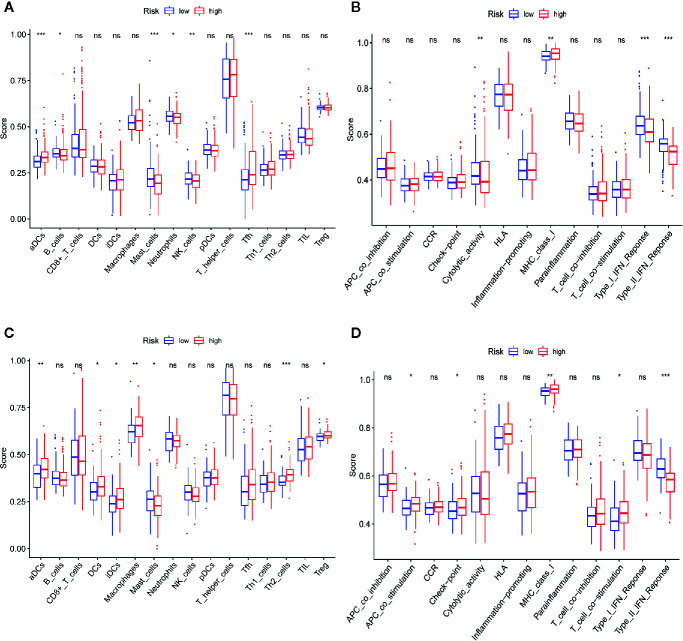
Comparison of single-sample gene set enrichment (ssGSEA) scores between high-risk and low-risk groups in TCGA-LIHC and ICGC-LIRI-JP datasets. **(A, B)** The scores of 16 immune cells and 13 immune-related functions are displayed in boxplots in TCGA-LIHC dataset. **(C, D)** The scores of 16 immune cells and 13 immune-related functions are displayed in boxplots in ICGC-RI-JP dataset. Adjusted P values are showed as: ns, not significant; *P < 0.05; **P < 0.01; ***P < 0.001.

To explore the correlation between the risk score and immune status, we calculated the infiltrating scores of 16 immune cells and 13 immune-related functions or pathways using ssGSEA. The scores of activated dendritic cells (aDCs), mast cells and follicular helper cells (Tfhs) were notably different between the high- and low-risk groups in the TCGA-LIHC dataset (all adj. P<0.001, **Figure 11A**). In the TCGA-LIHC dataset, the scores of cytolytic activity, type I interferon (IFN) response and type II IFN response were obviously higher in the low-risk group, while the score of MHC class I was lower in the low-risk group (all adj. P<0.01, **Figure 11B**). Moreover, the ICGC-LIRI-JP dataset showed that aDCs, mast cells, MHC class I and type II IFN responses were significantly different between the two risk cohorts (**Figures 11C, D**), which is consistent with the results of the TCGA-LIHC dataset.

## Discussion

As a common solid-tumor malignancy with high mortality, HCC has brought great socioeconomic pressure to HCC patients and their families. Owing to the complex etiological factors and high heterogeneity of HCC, it remains difficult to accurately predict the survival of HCC patients. Thus, it is urgent to detect effective prognostic biomarkers to monitor the progression and predict the prognosis of HCC patients. In this study, 393 differentially coexpressed genes were obtained through WGCNA and differential gene expression analysis. Then, these genes were used to construct a PPI network, and 38 hub genes were observed to be closely correlated with OS. Subsequently, we established a novel four-gene prognostic signature in the TCGA-LIHC dataset and built a nomogram based on this novel module, which showed acceptable accuracy and calibration. Afterward, the four-gene signature module was verified in the TCGA-LIHC dataset using the LASSO algorithm. To improve the robustness of the signature, we used the ICGC-LIRI-JP dataset for further validation. The four-gene signature was still found to have independent prognostic value. Finally, ssGSEA revealed significant differences in aDCs, mast cells, MHC class I and type II IFN responses between the two risk cohorts.

Several prior analyses have also shown that certain gene signatures may predict patient survival (20–26); however, our study has some differences and/or advantages compared with similar analyses. First, the gene signatures built in previous studies require many genes (20–23), which possibly leads to some difficulties in real-world practice. Our novel signature requires only 4 genes, and the predictive ability of our signature is acceptable, which increases the feasibility of the use of our signature in real-world practice. Second, in our study, we simultaneously used WGCNA, differential gene expression analysis, PPI network construction, univariate Cox regression analysis and LASSO Cox regression analysis, and these methods were rarely used together in one study for the construction of a prognostic module of HCC, which is a novel point of our study. Third, some previous studies did not verify their gene signature (24–26) using other datasets; however, we used two datasets (the ICGC-LIRI-JP dataset and GSE112790) for external validation, which is helpful to enhance the reliability of our findings. Interestingly, we observed that most differentially coexpressed hub genes (38/40) were significantly associated with survival time according to the results of the univariate Cox regression analysis. This finding suggests the possibility of establishing a prognostic signature using these differentially coexpressed hub genes.

The prognostic module proposed in our analysis was composed of CDCA8, KIF20A, KIF2C and CEP55, all of which are often reported as being dysregulated in HCC tissues (27–30). First, cell division cycle associated 8 (CDCA8) is regarded as a significant oncogene that is involved in the pathological development of various cancers, including HCC (27) and pancreatic ductal adenocarcinoma (31). Wu et al. reported that CDCA8 is obviously overexpressed at the mRNA and protein levels in HCC tissues, and the authors validated this finding at the mRNA level using real-time quantitative PCR (RT-qPCR) (32).Similarly, CDCA8 is closely correlated with cell division and growth in HCC, and CDCA8 is strongly associated with the pathological grades and T stages of HCC (33). Second, kinesin family member 20A (KIF20A) and KIF2C are the members of the kinesin superfamily proteins, both of which are closely regulated by E2F1. The depletion of KIF20A or KIF2C results in deforming microtubule structures, influencing cell motility and inhibiting cancer metastasis (34). A recent study suggested that KIF20A and KIF2C are obviously upregulated in HCC tissues, and higher expression of KIF20A and KIF2C correlates with worse survival (including OS and disease-free survival [DFS]), higher tumor stages and poorer pathological grades (35). Moreover, by conducting basic experiments, this study also showed that the downregulation of KIF20A and KIF2C can effectively inhibit the proliferation of HCC cells and increase G1 arrest in HCC cells (35). In addition, Lu et al. observed that high KIF20A expression was associated with more high-grade HCC (52.3% vs. 32.5%, P=0.003), more advanced HCC (45.9% vs. 21.1%, P<0.0001), and more deaths (65.7% vs. 28.9%, P<0.0001) than low KIF20A expression, and the authors also reported that KIF20A could act as an independent prognostic indicator for poor OS (HR=1.30, 95% CI: 1.16–1.47, P<0.001) and recurrence-free survival (RFS) (HR=1.14, 95% CI: 1.03–1.27, P < 0.001) (36). KIF2C contributed to cell proliferation, adverse invasion, and metastasis *in vitro* and *in vivo* by performing both gain- and loss-of-function assays, and the authors further suggested that KIF2C plays an important role in mediating the crosstalk between Wnt/β-catenin and mammalian target of rapamycin complex 1 (mTORC1) signaling in the pathogenesis of HCC (37). Third, centrosomal protein 55 (CEP55) contributes to the carcinogenesis of many cancers and regulates PI3K/AKT signaling (38). Yang et al. showed that CEP55 is upregulated in HCC tissues, and CEP55 overexpression correlates with poor tumor grades and high T stages; the authors also showed that CEP55 acts as an independent predictor of the OS of HCC patients using multivariate analysis (39). In addition, CEP55 was found to promote cell migration and adverse invasion *via* the regulation of the JAK2-STAT3-MMP signaling pathway in HCC, and the knockdown of CEP55 strongly suppressed HCC cell migration and invasion (40).

Several limitations to our analysis exist. 1) The TCGA-LIHC dataset provides multiple HCC tissue samples, and the ICGC-LIRI-JP dataset and GSE112790 were applied for external validation. However, these datasets were obtained from public databases, and additional real-world datasets are required to validate the clinical utility of the four-gene prognostic signature. 2) Although we utilized comprehensive bioinformatics approaches to construct and validate this prognostic signature in HCC, it may not be very accurate for HCC patients with different grades and stages. 3) We did not verify the correlation between the risk score and immune status by conducting basic experiments, which is a significant issue that deserves further investigation in the future.

## Conclusion

This comprehensive analysis proposes a novel prognostic signature of four differentially coexpressed hub genes that has satisfactory prognostic value. This model was an independent predictor of OS in the TCGA-LIHC and ICGC-LIRI-JP datasets, providing insight into the prediction of HCC prognosis. Nevertheless, additional studies are required to further explore the underlying mechanisms of these differentially coexpressed hub genes and tumor immunity.

## Data Availability Statement

The original contributions presented in the study are included in the article/**Supplementary Material**. Further inquiries can be directed to the corresponding author.

## Author Contributions

ZM had full access to all of the data in the manuscript and takes responsibility for the integrity of the data and the accuracy of the data analysis. Concept and design: All authors. Acquisition, analysis, and interpretation of data: ZM. Drafting of the manuscript: ZM. Critical revision of the manuscript for important intellectual content: All authors. Statistical analysis: All authors. Supervision: All authors. All authors contributed to the article and approved the submitted version.

## Funding

This study is supported by the Public Welfare Technology Application Research Program of Huzhou (No. 2019GY35, 2019GY01) and Young Talents Project of Huzhou Central Hospital (NO. 2020YC09), without the involvement of commercial entities. The funder had no role in the design or performance of the study; the collection, management, analysis, and interpretation of the data; the preparation, review, and approval of the manuscript; or the decision to submit the manuscript for publication.

## Conflict of Interest

The authors declare that the research was conducted in the absence of any commercial or financial relationships that could be construed as a potential conflict of interest.

## Supplementary Material

Supplementary Figure 1 Functional enrichment analysis of 40 differentially co-expressed genes. **(A)** Gene ontology (GO) enrichment analysis of 40 differentially co-expressed genes. **(B)** Kyoto encyclopedia of genes and genomes (KEGG) pathway analysis of 40 differentially co-expressed genes.

Supplementary Figure 2 Validation of the expression patterns of the four genes in the prognostic signature between LIHC and non-tumorous tissues based on GSE112790. The expression pattern of **(A)** CDCA8, **(B)** KIF20A, **(C)** KIF2C, and **(D)** CEP55 between LIHC and non-tumorous tissues.

Supplementary Figure 3 Scree plot of the principal component analysis (PCA) in TCGA-LIHC and ICGC-LIRI-JP datasets. **(A)** In the PCA of the TCGA-LIHC dataset, the first principal component (PC1) could explain 88.6% of total variance, while the second principal component (PC2) could explain 5.4% total variance. **(B)** In the PCA of the ICGC-LIRI-JP dataset, the PC1 could explain 79% of total variance, whereas the PC2 could explain 13% total variance.

Supplementary Table 1 The co-expression genes in the yellow module of TCGA-LIHC dataset.

Supplementary Table 2 The differentially expressed genes (DEGs) in TCGA-LIHC dataset.

Supplementary Table 3 Degree scores of all differential co-expressed genes.

Supplementary Table 4 DEGs between high-risk and low-risk cohorts in TCGA-LIHC dataset.

Supplementary Table 5 DEGs between high-risk and low-risk cohorts in ICGC-LIRI-JP dataset.

## Abbreviations

HCC, hepatocellular carcinoma; HBV, hepatitis B virus; HCV, hepatitis C virus; WGCNA, Weighed Gene Co-expression Network Analysis; DEGs, differentially expressed genes; TCGA, The Cancer Genome Atlas; ICGC, International Cancer Genome Consortium; ssGSEA, single-sample Gene Set Enrichment Analysis; LIHC, liver hepatocellular carcinoma; PPI, protein-protein interaction network; FDR, false discovery Rate; BH, Benjamini–Hochberg; TOM, topological overlap matrix; CPM, count per million; GO, gene ontology; KEGG, kyoto encyclopedia of genes and genomes pathway; STRING, Search Tool for the Retrieval of Interacting Genes; OS, overall survival; HR, hazard ratio; CI, confidence interval; LASSO, the least absolute shrinkage and selection operator analysis; ROC, receiver operating characteristic curves; PCA, principal component analysis; PC1, the first principal component; PC2, the second principal component; t-SNE, t-distributed stochastic neighbor embedding; tSNE1, the first component of t-SNE; tSNE2, the second component of t-SNE; AUC, Area Under Curve; aDCs, activated dendritic cells; IFN, interferon; DCs, dendritic cells; iDCs, immature Dendritic Cells; pDCs, plasmacytoid dendritic cells; NK cells, natural killer cells; Tfhs, follicular helper cells; Th1 cells, helper one T-cells; Th2 cells, helper two T-cells; TIL, tumor infiltrating lymphocytes; Treg, regulatory T cells; APC, antigen presenting cell; CCR, chemokine receptor type; HLA, human leukocyte antigen; MHC, major histocompatibility complex; RT-qPCR, real-time quantitative PCR; DFS, disease-free survival; RFS, recurrence-free survival; mTORC1, mammalian target of rapamycin complex1 signaling.

## References

[1] Siegel RL Miller KD Jemal A. Cancer statistics, 2020. CA Cancer J Clin (2020) 70(1):7–30. 10.3322/caac.21590 31912902

[2] Vogel A Saborowski A. Current strategies for the treatment of intermediate and advanced hepatocellular carcinoma. Cancer Treat (2020) 82:101946. 10.1016/j.ctrv.2019.101946 31830641

[3] Kole C Charalampakis N Tsakatikas S Vailas M Moris D Gkotsis E. Immunotherapy for Hepatocellular Carcinoma: A 2021 Update. Cancers (Basel) (2020) 12(10):E2859. 10.3390/cancers12102859 33020428 PMC7600093

[4] Caines A Selim R Salgia R. The Changing Global Epidemiology of Hepatocellular Carcinoma. Clin Liver Dis (2020) 24(4):535–47. 10.1016/j.cld.2020.06.001 33012444

[5] Huo J Wu L Zang Y. A robust nine-gene prognostic signature associated with tumour doubling time for hepatocellular carcinoma. Life Sci (2020) 260:118396. 10.1016/j.lfs.2020.118396 32918973

[6] Wang Z Zhu J Liu Y Liu C Wang W Chen F. Development and validation of a novel immune-related prognostic model in hepatocellular carcinoma. J Transl Med (2020) 18(1):67. 10.1186/s12967-020-02255-6 32046766 PMC7011553

[7] Morganti S Tarantino P Ferraro E D'Amico P Duso BA Curigliano G. Next Generation Sequencing (NGS): A Revolutionary Technology in Pharmacogenomics and Personalized Medicine in Cancer. Adv Exp Med Biol (2019) 1168:9–30 . 10.1007/978-3-030-24100-1_2 31713162

[8] Nangraj AS Selvaraj G Kaliamurthi S Kaushik AC Cho WC Wei DQ. Integrated PPI- and WGCNA-Retrieval of Hub Gene Signatures Shared Between Barrett’s Esophagus and Esophageal Adenocarcinoma. Front Pharmacol (2020) 11:881. 10.3389/fphar.2020.00881 32903837 PMC7438937

[9] Zhou Q Zhou LQ Li SH Yuan YW Liu L Wang JL. Identification of subtype-specific genes signature by WGCNA for prognostic prediction in diffuse type gastric cancer. Aging (Albany NY) (2020) 12(17):17418–35. 10.18632/aging.103743 PMC7521533 32915770

[10] Reddy RRS Ramanujam MV. High Throughput Sequencing-Based Approaches for Gene Expression Analysis. Methods Mol Biol (2018) 1783:299–323. 10.1007/978-1-4939-7834-2_15 29767369

[11] Robinson MD McCarthy DJ Smyth GK. edgeR: a Bioconductor package for differential expression analysis of digital gene expression data. Bioinformatics (2010) 26(1):139–40. 10.1093/bioinformatics/btp616 PMC2796818 19910308

[12] Fujimoto A Furuta M Totoki Y Tsunoda T Kato M Shiraishi Y. Whole-genome mutational landscape and characterization of noncoding and structural mutations in liver cancer. Nat Genet (2016) 48(5):500–9. 10.1038/ng.3547 27064257

[13] Ritchie ME Phipson B Wu D Hu Y Law CW Shi W. limma powers differential expression analyses for RNA-sequencing and microarray studies. Nucleic Acids Res (2015) 43(7):e47. 10.1093/nar/gkv007 25605792 PMC4402510

[14] Franceschini A Szklarczyk D Frankild S Kuhn M Simonovic M Roth A. STRING v9.1: Protein-protein interaction networks, with increased coverage and integration. Nucleic Acids Res (2013) 41(Database issue):D808–15. 10.1093/nar/gks1094 PMC3531103 23203871

[15] Smoot ME Ono K Ruscheinski J Wang PL Ideker T. Cytoscape 2.8: New features for data integration and network visualization. Bioinformatics (2011) 27(3):431–2. 10.1093/bioinformatics/btq675 PMC3031041 21149340

[16] Chin CH Chen SH Wu HH Ho CW Ko MT Lin CY. cytoHubba: identifying hub objects and sub-networks from complex interactome. BMC Syst Biol (2014) 8 Suppl 4(Suppl 4):S11. 10.1186/1752-0509-8-S4-S11 25521941 PMC4290687

[17] Simon N Friedman J Hastie T Tibshirani R. Regularization Paths for Cox’s Proportional Hazards Model via Coordinate Descent. J Stat Software (2011) 39(5):1–13. 10.18637/jss.v039.i05 PMC4824408 27065756

[18] Belhechmi S Bin R Rotolo F Michiels S. Accounting for grouped predictor variables or pathways in high-dimensional penalized Cox regression models. BMC Bioinf (2020) 21(1):277. 10.1186/s12859-020-03618-y PMC7331150 32615919

[19] Yi M Nissley DV McCormick F Stephens RM. ssGSEA score-based Ras dependency indexes derived from gene expression data reveal potential Ras addiction mechanisms with possible clinical implications. Sci Rep (2020) 10(1):10258. 10.1038/s41598-020-66986-8 32581224 PMC7314760

[20] Huo J Wu L Zang Y. A Prognostic Model of 15 Immune-Related Gene Pairs Associated With Tumor Mutation Burden for Hepatocellular Carcinoma. Front Mol Biosci (2020) 7:581354. 10.3389/fmolb.2020.581354 33282911 PMC7691640

[21] Zhu Z Li L Xu J Ye W Chen B Zeng J. Comprehensive analysis reveals a metabolic ten-gene signature in hepatocellular carcinoma. PeerJ (2020) 8:e9201. 10.7717/peerj.9201 32518728 PMC7258935

[22] Ouyang G Yi B Pan G Chen X. A robust twelve-gene signature for prognosis prediction of hepatocellular carcinoma. Cancer Cell Int (2020) 20:207. 10.1186/s12935-020-01294-9 32514252 PMC7268417

[23] Xie H Liu S Zhang Z Chen P Tao Y. A novel seven-gene signature as Prognostic Biomarker in Hepatocellular Carcinoma. J Cancer (2020) 11(19):5768–81. 10.7150/jca.44573 PMC7477431 32913470

[24] Dong S Lu LJ. An alternative splicing signature model for predicting hepatocellular carcinoma-specific survival. J Gastrointest Oncol (2020) 11(5):1054–64. 10.21037/jgo-20-377 PMC7657838 33209497

[25] Wang J Miao Y Ran J Yang Y Guan Q Mi D. Construction prognosis model based on autophagy-related gene signatures in hepatocellular carcinoma. Biomark Med (2020) 14(13):1229–42. 10.2217/bmm-2020-0170 33021390

[26] Wu ZH Yang DL. Identification of a protein signature for predicting overall survival of hepatocellular carcinoma: a study based on data mining. BMC Cancer (2020) 20(1):720. 10.1186/s12885-020-07229-x 32746792 PMC7398333

[27] Zhang B Tang B Gao J Li J Kong L Qin L. A hypoxia-related signature for clinically predicting diagnosis, prognosis and immune microenvironment of hepatocellular carcinoma patients. J Transl Med (2020) 18(1):342. 10.1186/s12967-020-02492-9 32887635 PMC7487492

[28] Liu J Lu J Ma Z Li W. A Nomogram Based on a Three-Gene Signature Derived from AATF Coexpressed Genes Predicts Overall Survival of Hepatocellular Carcinoma Patients. BioMed Res Int (2020) 2020:7310768. 10.1155/2020/7310768 32382568 PMC7195644

[29] Ji Y Yin Y Zhang W. Integrated Bioinformatic Analysis Identifies Networks and Promising Biomarkers for Hepatitis B Virus-Related Hepatocellular Carcinoma. Int J Genomics (2020) 2020:2061024. 10.1155/2020/2061024 32775402 PMC7407030

[30] Xiang XH Yang L Zhang X. Seven-senescence-associated gene signature predicts overall survival for Asian patients with hepatocellular carcinoma. World J Gastroenterol (2019) 25(14):1715–28. 10.3748/wjg.v25.i14.1715 PMC6465944 31011256

[31] Li B Liu B Zhang X Liu H He L. KIF18B promotes the proliferation of pancreatic ductal adenocarcinoma via activating the expression of CDCA8. J Cell Physiol (2020) 235(5):4227–38. 10.1002/jcp.29201 31875977

[32] Wu B Huang Y Luo Y Ma A Wu Z Gan Y. The diagnostic and prognostic value of cell division cycle associated gene family in Hepatocellular Carcinoma. J Cancer (2020) 11(19):5727–37. 10.7150/jca.46554 PMC7477449 32913466

[33] Xu D Liu X Wang Y Zhou K Wu J Chen JC. Identification of immune subtypes and prognosis of hepatocellular carcinoma based on immune checkpoint gene expression profile. Biomed Pharmacother (2020) 126:109903. 10.1016/j.biopha.2020.109903 32113055

[34] Jung YD Cho JH Park S Kang M Park SJ Choi DH. Lactate Activates the E2F Pathway to Promote Cell Motility by Up-Regulating Microtubule Modulating Genes. Cancers (Basel) (2019) 11(3):274. 10.3390/cancers11030274 PMC6468617 30813560

[35] Chen J Li S Zhou S Cao S Lou Y Shen H. Kinesin superfamily protein expression and its association with progression and prognosis in hepatocellular carcinoma. J Cancer Res Ther (2017) 13(4):651–9. 10.4103/jcrt.JCRT_491_17 28901309

[36] Lu M Huang X Chen Y Fu Y Xu C Xiang W. Aberrant KIF20A expression might independently predict poor overall survival and recurrence-free survival of hepatocellular carcinoma. IUBMB Life (2018) 70(4):328–35. 10.1002/iub.1726 29500859

[37] Zhang GP Shen SL Yu Y Yue X Hu WJ Li SQ. Kinesin family member 2C aggravates the progression of hepatocellular carcinoma and interacts with competing endogenous RNA. J Cell Biochem (2020) 121(11):4419–30. 10.1002/jcb.29665 32056305

[38] Jeffery J Sinha D Srihari S Kalimutho M Khanna KK. Beyond cytokinesis: the emerging roles of CEP55 in tumorigenesis. Oncogene (2016) 35(6):683–90. 10.1038/onc.2015.128 25915844

[39] Yang L He Y Zhang Z Wang W. Upregulation of CEP55 Predicts Dismal Prognosis in Patients with Liver Cancer. BioMed Res Int (2020) 2020:4139320. 10.1155/2020/4139320 32337246 PMC7153005

[40] Li M Gao J Li D Yin Y. CEP55 Promotes Cell Motility via JAK2-STAT3-MMPs Cascade in Hepatocellular Carcinoma. Cells (2018) 7(8):99. 10.3390/cells7080099 PMC6115913 30096813

