# Supplementary figures and images for: Identification of a Novel Four-Gene Signature Correlated With the Prognosis of Patients With Hepatocellular Carcinoma: A Comprehensive Analysis
Source: Front Oncol. 2021 Mar 12;11:626654. doi: 10.3389/fonc.2021.626654 (PMC7994902; doi:10.3389/fonc.2021.626654)

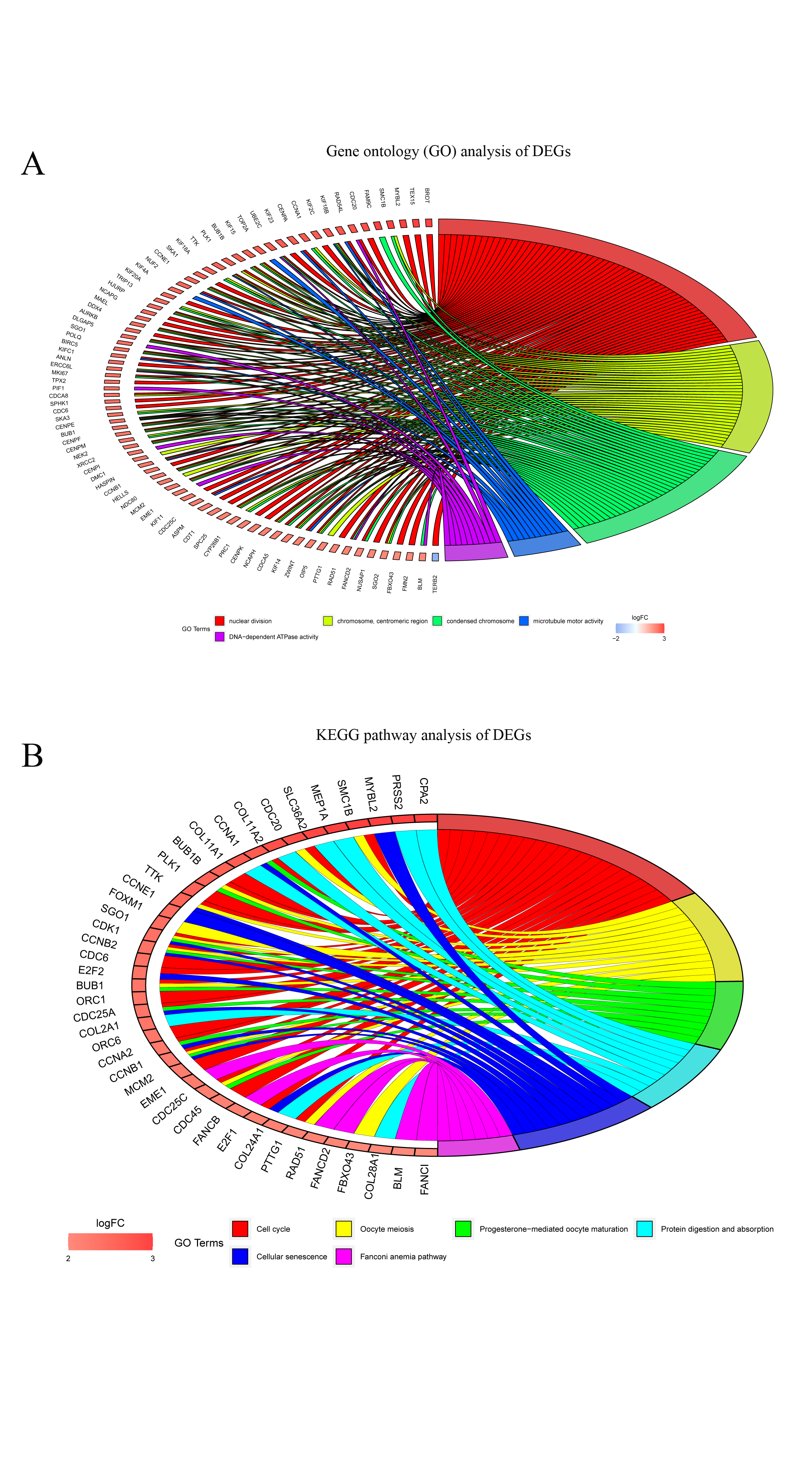

Supplement: Supplementary Figure 1 — Functional enrichment analysis of 40 differentially co-expressed genes. (A) Gene ontology (GO) enrichment analysis of 40 differentially co-expressed genes. (B) Kyoto encyclopedia of genes and genomes (KEGG) pathway analysis of 40 differentially co-expressed genes. [file Image_1.tif]

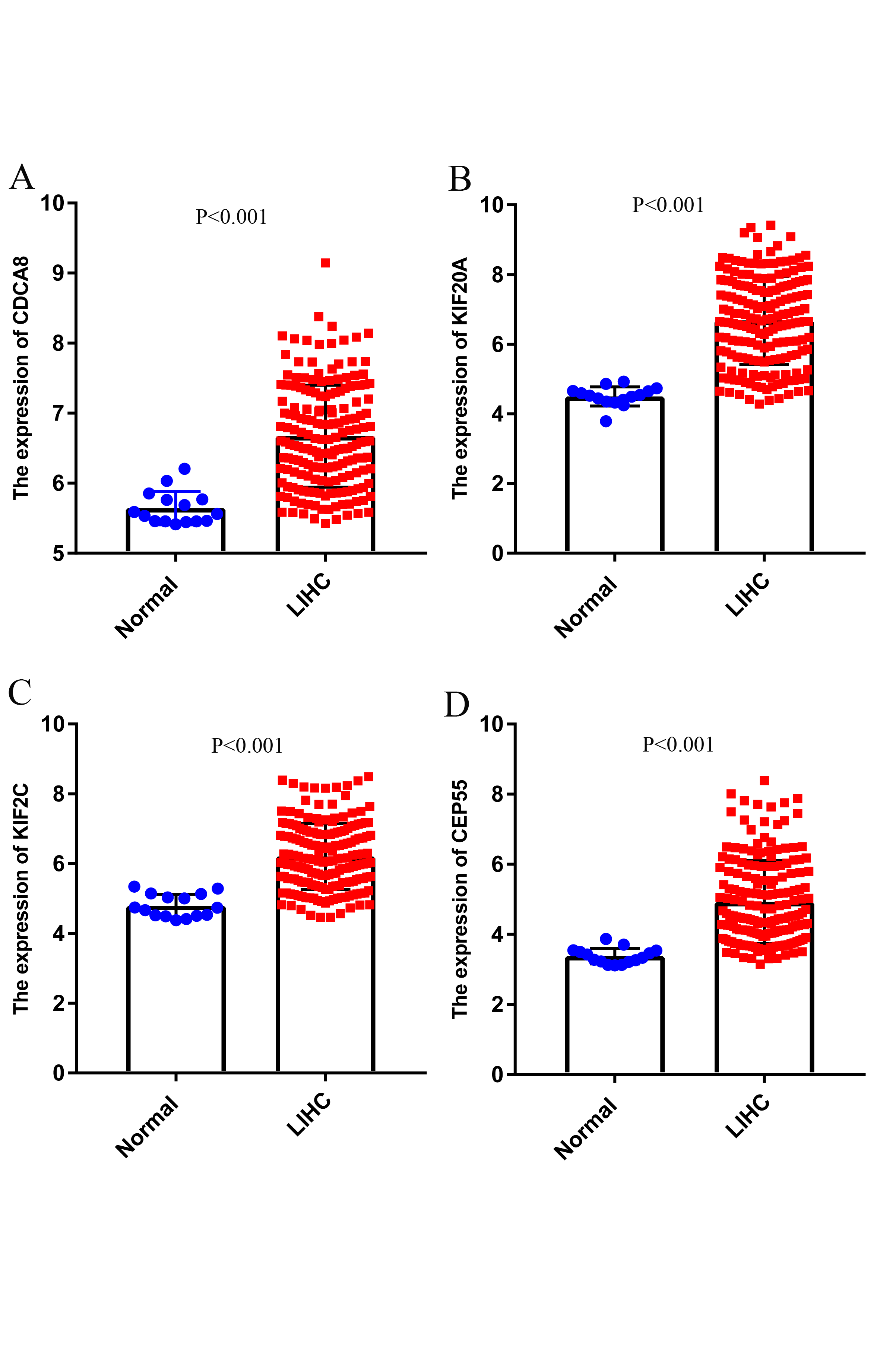

Supplement: Supplementary Figure 2 — Validation of the expression patterns of the four genes in the prognostic signature between LIHC and non-tumorous tissues based on GSE112790. The expression pattern of (A) CDCA8, (B) KIF20A, (C) KIF2C, and (D) CEP55 between LIHC and non-tumorous tissues. [file Image_2.tif]

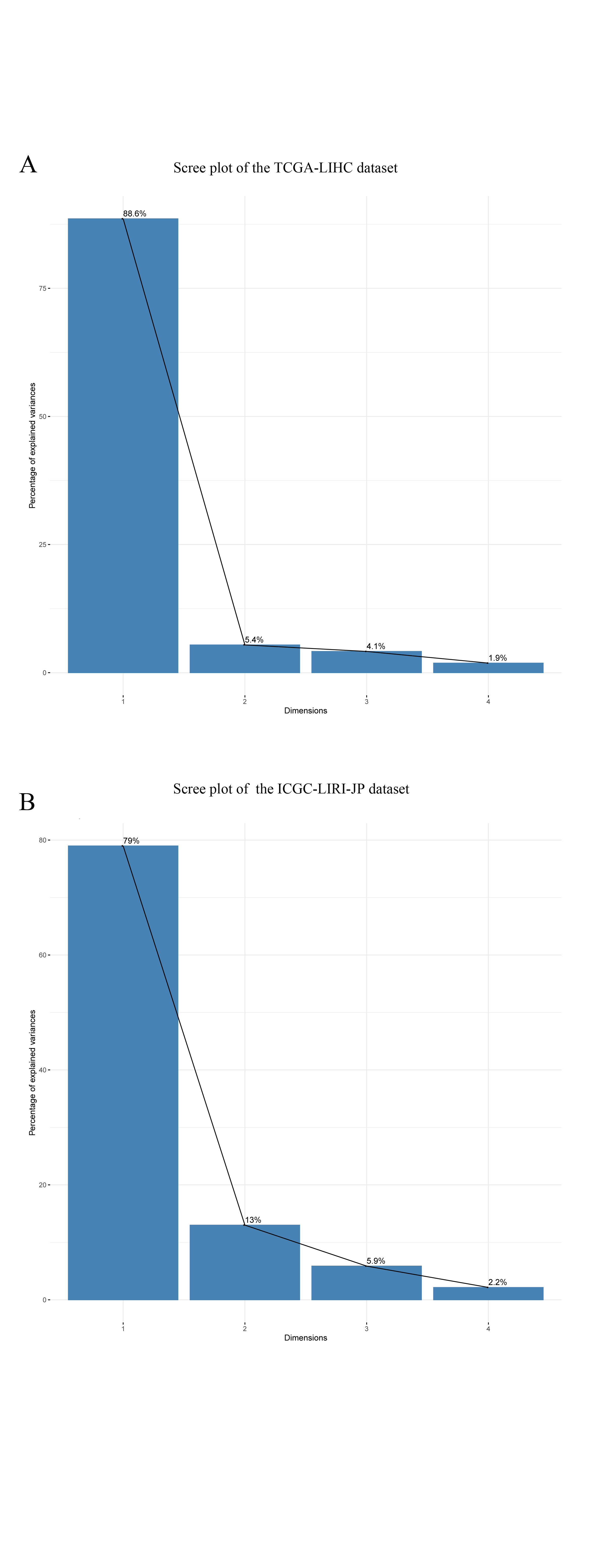

Supplement: Supplementary Figure 3 — Scree plot of the principal component analysis (PCA) in TCGA-LIHC and ICGC-LIRI-JP datasets. (A) In the PCA of the TCGA-LIHC dataset, the first principal component (PC1) could explain 88.6% of total variance, while the second principal component (PC2) could explain 5.4% total variance. (B) In the PCA of the ICGC-LIRI-JP dataset, the PC1 could explain 79% of total variance, whereas the PC2 could explain 13% total variance. [file Image_3.tif]
